# Response of Healthcare Workers to COVID-19 Protocols after the Index Case at 37 Military Hospital, Ghana

**DOI:** 10.1155/2021/2873859

**Published:** 2021-04-27

**Authors:** D. Afeng-Nkansah, E. Asumanu, P. Nyinaku, F. Acheampong, R. Lamptey

**Affiliations:** ^1^Medical Division, 37 Military Hospital, Ghana; ^2^Postgraduate Unit, 37 Military Hospital, Ghana; ^3^Public Health Division, 37 Military Hospital, Ghana; ^4^Research Unit, Korle-Bu Teaching Hospital, Ghana; ^5^Department of Family Medicine, Korle-Bu Teaching Hospital, Ghana

## Abstract

The diagnosis and management of COVID-19 are much dependent on the adherence to standardized protocols. Healthcare workers play a crucial role in the case management of COVID-19 in many institutions. Globally, the disease burden is increasing, and the mortality has reached over 2 041 426 compared with 323 000 in May 2020. In West Africa, the pandemic has shown a slow but steady rise in many countries. Existing protocols and their utilization are best assessed after the occurrence of the index case. *General aim*. The study assessed the health worker's response to COVID-19 protocols at three designated areas of the in-hospital management care triaging, holding area, and treatment centers. *Method*. A qualitative design was used to assess the response of healthcare workers with regards to early case detection, infection prevention, risk communication to clients and compliance to protocols. The study conducted observational visits and purposively selected healthcare workers comprising of clinicians, nurses, emergency medical technicians, and laboratory technicians who perform routine duties at the triaging, holding, and treatment centers. A total of 41 observations were made over two weeks. *Results*. Participants comprised 23 males and 18 females. At all observed units, the case definition was being used to screen attendants presenting, and appropriate categorization of patients was ensured. The use of temperature in screening for COVID-19 at the units was generally adhered to. Only 50% of participants used the prescribed PPEs. The physical distancing between healthcare workers and client and between clients and caregivers were not enforced; however, hand hygiene was practiced. Disinfection of working surfaces and equipment with 0.5% chlorine or 70% alcohol-based rubs were used most of the time. It was observed however that no psychological counselling was given to suspected cases or their relatives. *Conclusion*. Healthcare workers showed discordant response to different parts of the protocols for COVID-19 especially appropriate distancing. There was an enhanced awareness among healthcare workers and improvement in infection prevention protocols. The study also observed that as the risk of infection increased from triaging to holding area and to treatment centers, the response of healthcare workers to COVID-19 protocols also improved. Risk communication is an essential part of the COVID-19 management strategy. At the treatment centers, healthcare workers adhered to this protocol, whereas it was a major gap at the triaging and holding areas.

## 1. Introduction

### 1.1. Background

Coronavirus disease 2019 (COVID-19) is a potentially severe acute respiratory infection caused by severe acute respiratory syndrome coronavirus 2 (SARS-CoV-2) [1]. The clinical presentation is that of respiratory infection with symptoms ranging from a mild common cold-like illness to severe viral pneumonia which may lead to acute respiratory distress syndrome and other systemic manifestations that are potentially fatal. COVID-19 may also be asymptomatic. The majority of the cases are asymptomatic or show mild symptoms. Common symptoms at presentations are fever, fatigue, cough (with or without sputum production), anorexia, malaise, muscle pain, sore throat, dyspnoea, nasal congestion, headache, and loss in sense of taste and/or smell [2]. The disease burden related to COVID-19 is high and rising globally. The outbreak escalated rapidly, and W.H.O. declared it as a public health emergency of international concern on 30^th^ January 2020 and later declared it a global pandemic on 11^th^ March 2020 [3]. The global prevalence as of January 2021 was 95 567 167 with 2 041 426 deaths compared with a global prevalence of 4 893 186 of 323 256 deaths as of 22^nd^ May 2020 [4, 5]. In Africa, a total of 3 280 896 confirmed cases with 79 088 deaths as of January 2021 compared with a total of 99 977 confirmed cases with 3095 death [5]. There are global concerns about the impact of COVID-19 in Africa and how the continent will manage the pandemic. Healthcare worker's response will be critical in the successful management of the COVID-19 pandemic in Africa. The next step in the containment of the crisis is the clinical case management by healthcare workers, and this requires adequate preparedness and timely response [6]. In Ghana, there are a total of 58 065 confirmed cases with 351deaths as of January 2021 compared with 6 486 cases with 31deaths in May 2020 [5]. There are national and institutional protocols for clinical case management. Three categories of healthcare workers are involved in the inhospital management of COVID-19 cases. These are healthcare workers at the triaging points, the holding area for suspected cases, and the treatment centers for confirmed cases. On 20th March 2020, the index case at the 37 Military Hospital presented with atypical symptoms of fever and abdominal pain which was followed by a 24-hour history of respiratory symptoms leading to the suspicion of COVID-19. Although there was a preparedness in place to handle COVID-19, the index case brought to bear the need to assess the effectiveness of detecting, isolating, and managing COVID-19 cases. This was in an anticipation of an increase in the number of cases. The general aim of this study was to assess the response of the healthcare workers of the 37 Military Hospital to adherence to the protocols in diagnosing COVID-19.

## 2. Materials and Methods

The study was a qualitative cross-sectional design to describe the response of the healthcare workers in the three critical aspects of patient triaging, holding units, and treatment centers at the 37 Military Hospital. This facility is now designated as a COVID-19 treatment center and receives referrals from peripheral sites. After the index case in Ghana, the hospital established the triaging system made up of triaging units at all entry and exit points. The hospital also created an UN-Level 1 Hospital as a holding area for suspected cases and also deployed an UN-Level II treatment center for COVID-19 cases [7]. There are 3 response teams made up of about 200 frontline health workers comprising of clinicians, intensive care staff, nurses, and other allied health workers. An average number of 982 patients attend the hospital daily. The triage unit is managed by a team of nurses and emergency medical technicians. The holding unit is managed by the clinicians, intensive care staff, nurses, and other allied health workers. The treatment center is managed by clinicians, intensive care staff, nurses, and other allied health workers.

The study conducted observational visits and purposively selected healthcare workers comprising of clinicians, nurses, emergency medical technician, and laboratory technicians who perform routine duties at the triaging, holding, and treatment centers. The study was carried out within two weeks from 9^th^ May 2020 to 23^rd^ May 2020 to highlight the healthcare worker's response after the index case in the hospital.

### 2.1. Data Collection Tool and Technique

An ethnographic study of the health worker's response, at the triaging areas, holding, and treatment centers was conducted. There were two observational visits on three 3 different days of the week for the two-week duration. Each investigator spent at most thirty minutes at each designated unit. A standardized checklist was used to collect the data, and observational notes were collected. The returned checklists were checked for completeness, and qualitative data was analyzed using NVivo software. This software was used to analyze the compliance to protocols among the three categories of healthcare workers at the different designated COVID-19 units.

### 2.2. Assessment Criteria

A predesigned format was used to assess the observation and performance of the frontline health workers.

The response was graded as

(A) Complete—proper and appropriate adherence to protocols for the designated areas
  - The triaging completeness included questioning clients about fever, cough, sneezing, difficulty in breathing, travel history, temperature check and appropriate physical distancing, hand hygiene, and use of recommended personal protective equipment and counselling
  - For the holding unit, completeness included questioning clients about fever, cough, sneezing, difficulty in breathing, travel history, temperature check and appropriate physical distancing, hand hygiene and use of recommended personal protective equipment, appropriate disinfection in a chlorine bath, breaking of news and counselling, and appropriate diagnosis
  - For the treatment center completeness included appropriate physical distancing, hand hygiene and use of recommended personal protective equipment, appropriate disinfection in a chlorine bath, adherence to allowable contact time in the red zones, appropriate handling of fomites, and psychotherapy
(B) Incomplete—where there exist gaps of inadequate use of protocol appropriate for the designated site
(C) Poor—lack of use of the protocol in designated areas

### 2.3. Ethical Approval and Confidentiality

Approval for the study was obtained from the 37 Military Hospital Institutional Review Board with reference-37MH-IRB IPN/FP/393/2020, Accra. Permission was granted by unit heads before data collection. The investigators were the only people who had access to the research data. Anonymity was ensured in the dissemination of findings from this study since participants were not identified by their names. The researchers ensured that whatever information they collected was handled with strict confidentiality and was entirely used for research purposes. Respondent's name or personal identifying information was not collected nor published in any report. The data was stored with passwords on electronic media and in safe locked boxes.

## 3. Results

A total of 41 observations were conducted over a two-week duration for health workers operating at the different areas of the COVID-19 response. The study results were analyzed using the categories of health worker response at the triaging, holding, and treatment centers for COVID-19.  Table 1 presents a summary of observations made during the study.

The study outcomes are captured under three main thematic areas. This is made up of (1) early detection of COVID-19 infection which consists of screening using a questionnaire and temperature checks. (2) Infection prevention among healthcare providers was investigated by observing the use of personal protective equipment (PPE), hand hygiene, physical distancing, and disinfection. (3) Risk communication including the breaking of news and psychological support offered to suspected and confirmed cases of COVID-19. The units studied are the triaging, holding, and treatment centers for COVID-19.

(1) Response health workers at triaging units
  - Early detection

The health workers adhered to the procedure for using the questionnaires as a screening tool at triaging, and holding areas were adhered to. All observations revealed that case definition was being used to screen attendants presenting at the unit, and appropriate categorization of patients was ensured. The use of temperature for screening for COVID-19 at the unit was generally adhered to. However, a few healthcare workers (4 out of 16 observations) did not use the appropriate technique in checking the temperature. During the period of observation, about 30 clients were screened.

(b) Infection prevention among healthcare workers

The use of appropriate PPE was generally not adhered to although perceived to be available. At the triaging areas, about 8 out of 16 observations representing 50% of healthcare workers did not use the prescribed PPEs. Some healthcare workers did not change their gloves after attending to clients. Hand hygiene was practized by all healthcare workers at the triaging. They used 0.05% chlorine in hand hygiene; however, the complete steps in the protocol for hand hygiene were not adhered to by the majority of participants.

The physical distancing between healthcare worker's and clients and clients and caregivers was not enforced. The majority of care providers about 73% did not enforce physical distancing. The enforcing physical distancing among the client and their care providers was poor. Disinfection of working surfaces and equipment with 0.5% chlorine or 70% alcohol-based rubs was observed to be undertaken in 60% of observations at the triaging area; however, a minimum of one disinfection per three sessions of attending patients was not observed.

(c) Risk communication to the client

At the triaging areas, it was observed that clients were not adequately informed about COVID-19. In 53% of observations, healthcare workers did not provide any information about COVID-19 to the clients whilst 40% were given some information. It was observed that no psychological counselling was given to suspected cases or their relatives.

(2) Response health workers at the holding area
  - Early detection of COVID-19

All observations revealed that a case-based form was used to screen attendants at the various emergencies and appropriate categorization of patients as either COVID-19 suspected or not suspected was ensured. Healthcare workers adhered to appropriate questioning, and complete filling of case-based forms as a screening tool at the holding area was adhered to. Routine temperature measurement was generally adhered to. However, a few healthcare providers (2 out of 17 observations) did not apply the appropriate technique in checking the temperature.

(b) Infection prevention among healthcare providers

At the holding area, 8 out of 17 representing 47% of observations revealed that healthcare workers did not use the appropriate PPE. It was observed that in the performance of various clinical activities the use of appropriate PPEs was lacking. However, there were reminders of protocols at the unit.

Hand hygiene was practized by all categories of healthcare providers at the holding unit for COVID-19. This included handwashing with soap under running water and the use of 70% alcohol-based sanitizers. During the study period, majority of participants adhered to the hand hygiene protocol.

The physical distancing between the healthcare provider and clients was not enforced. During observations, the majority of healthcare providers, 77%, did not enforce physical distancing policy at the holding area. This practice was more so among the client and their care providers.

Disinfection was performed during all observations at various stages of care delivery. This included disinfection of working surfaces, equipment, and removal of PPE such as gloves, coverall, gumshoes, cloaks, disposable aprons, linen, and fomites of patients. During most of the observation, the working surfaces were disinfected with 70% alcohol-based rubs after attending to each suspected case. Disinfection of working surfaces was performed in 95% of all observations at the holding area. All PPE was disinfected with 0.5% chlorine before disposal. Fomites of patients were decontaminated and disposed of appropriately.

(c) Risk communication

At the holding areas most clients, 14 out of 16 representing (87.5%) were not given adequate information about COVID-19. During the study period, about 38 clients were seen at this unit. No psychological counselling was observed to be offered to the suspected COVID-19 cases at the holding area.

(3) Response health workers at the treatment centre

All 24 patients who were on admission at the treatment centre were laboratory-confirmed COVID-19 cases.

(b) Infection prevention among healthcare providers

At the treatment center, most healthcare workers performed focused multitasked activities and reduced contact time with patients. All observations made at the treatment centre found all health workers performing various duties using appropriate PPEs; however, physical distancing was not adhered to. All PPEs were decontaminated using 0.5% chlorine before reuse or disposal. Regular disinfection of surfaces at the wards of the treatment center was observed; however, the restrooms and changing rooms and working surfaces were not disinfected frequently.

(c) Risk communication

Appropriate protocols of breaking of the news to patients were followed, and psychological support was provided for all patients on admission.

### 3.1. Compliance

Table 2 shows the overall compliance of the various units in the response of the healthcare workers.

The overall level of compliance at the different levels improved from the triaging to the holding and treatment areas. It was generally observed that as the risk of infection increased, the level of compliance increased.

## 4. Discussion

Healthcare worker's response to the COVID 19 pandemic has contributed to disease outcomes and containment. The best clinical management outcomes are dependent on the coordination of responses in triaging, holding, and treatment centers. Many countries in Africa are stepping up their preparedness to detect and cope with COVID-19 importations. Early detection of COVID-19 if enhanced will prevent importation as well as the horizontal spread of the disease [8, 9]. In this study, it was therefore imperative to interrogate the gaps in each area of the COVID 19 management.

### 4.1. Early Detection of COVID-19

In assessing protocols on early detection, screening of cases using the recommended questionnaire was essentially adhered to at the triaging and holding areas. All observations revealed that case definition was used to screen attendants, and appropriate categorization of patients was ensured [10]. This observation was key in the detection of suspected cases. However, the use of temperature as a screening tool for COVID-19 was not found to be an effective method for screening in this study; although, the temperature was found to be key in diagnosing COVID-19 as found in 98.6% of the population studied by Wong et al. [9]. A gap detected during this study was the lack of compliance by respondents at the triaging and holding areas to standardized technique and skill in measuring the temperature. Whereas the standardized questionnaire was efficiently used, suspected COVID-19 cases may be missed because the use of temperature in screening was lacking.

### 4.2. Infection Prevention and Control

The appropriate use of personal protective equipment (PPE) is crucial in the minimization of the risk of exposure. In the management of other contagious diseases such as Ebola, the suitable selection, use, and doffing of PPE are vital in reducing the risk of exposure. Studies show that adherence varies by type, clinical area, and availability of the PPE depicting the importance of PPE in COVID-19 management [11]. However, in this study, PPE usage at the triaging was unsatisfactory although PPEs were perceived to be available. It was evident that 8 out of 16 observations representing 50% of observations did not use the prescribed PPEs whilst attending patients. This observation was no different at the holding area; however, at the treatment center, the study found all performing various duties using appropriate PPEs. This finding confirms the evidence that the perception of risk of exposure will lead to adherence to the use of appropriate PPEs [12].

It was observed that hand hygiene was practized by all healthcare workers at the triaging, holding, and treatment centers for COVID-19. This was key in controlling transmission of infection [11, 12]; however, gaps existed in the hand hygiene protocols. Identified gaps such as infrequent washing of hands with soap under running water and use of alcohol-based hand sanitizer were observed at the triaging area.

Physical distancing described as a reduction of contact time with patients is critical in preventing exposure to COVID-19 [13, 14]. At the triaging and holding areas, a physical distancing between healthcare workers and clients and between clients and caregivers was not enforced. Center for Disease Control recommends a minimum of 6 feet between individuals but this was difficult to enforce because of the cultural context on the communities being served by the facility. Culturally, the caregiver is expected, to be with their sick relatives and will not distance themselves even when instructed to. This observation revealed the low level of awareness and adherence to protective measures among the general population on COVID-19. Observance of physical distancing among healthcare providers and caregivers was challenging; however, horizontal transmission of COVID-19 was reduced because of the other measures of infection prevention such as disinfection of working surfaces, decontaminated and appropriately disposed of fomites being practized, and the low prevalence in the community [15, 16]. At the treatment center, however, most healthcare providers performed focused multitasked activities and reduced contact time with patients.

Disinfection was performed during all observations. This included disinfecting working surfaces with 0.5% chlorine or 70% alcohol-based rubs. Disinfection of working surfaces was performed in 60% of observations at the triaging area and 95% at the holding area. Working surfaces in the wards of the treatment centre were not observed to be disinfected.

According to a study by Tvedt et al., disinfection is a robust hand hygiene method for infection prevention, and viruses can be killed on surfaces with disinfection [15, 17].

### 4.3. Risk Communication

Risk communication is an essential part of effective response and serves as a key public health intervention during epidemics and emergencies [18]. Clinician-patients communication is therapeutic. It provides comfort, alleviates patient's anxiety, and influences the health outcome through an indirect route. There is increased adherence, better self-care skills, and improvement in overall well-being [19].

Breaking bad news to patients is one of the most difficult task clinicians faced. Evidence from the literature suggests that patients are dissatisfied with the information they receive, and only a third of healthcare workers are competent with their interaction skills. Breaking bad news requires further attention to facilitate optimal care [20].

At the triaging and holding areas, it was observed that clients were not adequately informed about their risk with regards to COVID-19. At the triaging in 53% of observations, healthcare workers did not provide any information about COVID-19 to the clients whilst 40% were given some information. At the holding area, 14 out of 16 representing 87.5% did not receive adequate information about COVID-19, whilst at the treatment center, psychological support was provided for all patients on admission.

### 4.4. Compliance of Healthcare Workers to Protocols

The overall compliance to protocols at the holding area and treatment center was assessed as good while at the triaging unit was assessed to be poor in the first week of the observations but improved remarkably in the second week. This can be attributed to the risk perception by healthcare workers with continuous training on infection prevention. In a study by Robyn et al., it was stated that healthcare worker's response improved when the risk assessment was perceived as high [21]. This can be concluded in our observations made from triaging to the holding area and the COVID-19 treatment center.

The study duration was short which limited the observation of change in practice arising from continuous training and supportive supervision undertaken by the management of the hospital. This could be investigated further to determine the change in behavior which is beyond the scope of this study.

## 5. Conclusion

Healthcare professionals showed discordant response to different parts of the protocols for COVID-19 especially appropriate distancing which aids an increase in awareness among healthcare workers and the improvement in the adherence to infection prevention protocols. The study also observed that as the risk of infection increased from triaging to holding area and to treatment centers, the response of healthcare workers to COVID-19 protocols also improved. Risk communication is an essential part of the COVID-19 management strategy. At the treatment centers, healthcare workers adhered to this protocol, whereas it was a major gap at the triaging and holding areas.

## Figures and Tables

**Table 1:** Frequencies of observations at study sites.

Place of observation	Frequency of observation	Percentage representation
Response at triaging	16	39.0%
Response at holding area	17	41.5%
Response at treatment center	8	19.5%
Total	41	100%

**Table 2:** Level of compliance among participants.

Response	Total number of observations	Frequency of compliant participants	Percentage of compliance among participants
Triaging	16	9	56.25
Holding area	17	16	94.11
Treatment center	8	8	100.00

## Data Availability

The datasheet and observational notes are available on request from the Principal Investigator.
